# The effect of mindfulness‐based stress reduction on maternal anxiety and self‐efficacy: A randomized controlled trial

**DOI:** 10.1002/brb3.1561

**Published:** 2020-03-11

**Authors:** Masoomeh Zarenejad, Mansooreh Yazdkhasti, Mitra Rahimzadeh, Zahra Mehdizadeh Tourzani, Sara Esmaelzadeh‐Saeieh

**Affiliations:** ^1^ Student Research Committee Alborz University of Medical Sciences Karaj Iran; ^2^ Social Determinants of Health Research Center Alborz University of Medical Sciences Karaj Iran; ^3^ Reproductive health Department Alborz University of Medical Sciences Karaj Iran

**Keywords:** anxiety, mindfulness, self‐efficacy, stress

## Abstract

**Objective:**

The aim of the study was to assess the effect of mindfulness‐based stress reduction (MBSR) on anxiety and self‐efficacy in coping with childbirth.

**Material and Methods:**

This randomized controlled trial was conducted on 70 pregnant women in Abyek city of Qazvin province in Iran. The convenient sampling method was recruited. Samples were assigned to control and intervention groups using random blocks. In addition to routine care, individuals in the intervention group received 6 MBSR training sessions. The data gathering questionnaire in this study included mindfulness, Pregnancy‐Related Anxiety Questionnaire, and self‐efficacy in coping with childbirth questionnaire.

**Results:**

There was no statistically significant difference between the demographic characteristics in the control and intervention groups. The results of the analysis of variance (ANOVA) with repeated measures indicated the effect of time on the change in the total score of anxiety in the intervention group (*p* = .001). There was a significant difference between the two groups (*p* = .001). Also, the results of ANOVA with repeated measures showed that time had no impact on the score of self‐efficacy in delivery coping (*p* = 0/1) and that there was no significant difference between the two groups in this respect (*p* = .6).

**Conclusion:**

The result of this study showed that mindfulness reduces anxiety of pregnant mothers, and it is suggested that mindfulness programs be educated for healthcare providers and pregnant mothers to reduce maternal anxiety and improve pregnancy outcomes and delivery.

## Graphical abstract

Mindfulness‐based stress reduction on anxiety and self‐efficacy in coping with childbirth in pregnant women.

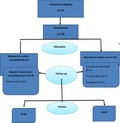

## 1 INTRODUCTION

Pregnancy and postpartum are associated with physical and mental changes and are the critical time for mothers to develop mental disorders (Forray, Focseneanu, Pittman, McDougle, & Epperson, 2010). Maternal stress during pregnancy causes preterm labor and low birthweight (Dunkel Schetter, 2011). Furthermore, it increases the negative effects on growth, development, cognition, and emotions during infancy and childhood (Polanska et al., 2017). The prevalence of anxiety has been reported 12.2%–39%, depending on the type of anxiety in pregnant women two or three times higher than the general population (Goodman et al., 2014). Pregnancy anxiety is a complex subjective response influenced by multifactors including gender (in women more than men), perceived risk for mother and child, social and family experiences, economic costs, child sex, and access to social support for pregnant women (Patel, Biros, Moore, & Miner, 2014). Studies have shown that improving mental health is associated with increased self‐efficacy (individual confidence in their ability to cope with special conditions) (Bandura, 1997), reduced fear of delivery, and improved delivery outcomes. Improvement of childbirth self‐efficacy is effective in reducing pain and is a preventive factor in the symptoms of post‐traumatic stress disorder after birth. On the other hand, self‐efficacy reduces physical and mental stress, which has shown an important links between mother's stress and child development (Byrne, Hauck, Fisher, Bayes, & Schutze, 2014; Farid & Akbari Kamrani, 2016). Due to changes during pregnancy, there is a need for psychological coping and response in pregnant women. Stress coping strategies involve the cognitive and behavioral effort that an individual uses for stressful situations (Manesh, Kalati, & Hosseini, 2015). Mindfulness‐based interventions have been identified as stress‐reducing and psychological improvement enhancers (Hughes et al., 2009). Mindfulness is the awareness that emerged through paying attention to a particular purpose, so that it is examined in the present moment without judging the experiences of the person (Taylor, Cavanagh, & Strauss, 2016). Kabat‐Zinn (1982) designed mindfulness‐based stress reduction (MBSR) program in 8‐week session which is the most well‐known mindfulness program (Creswell, 2017). The mindfulness increases activity of the cortical region in the frontal and reduced functional communication in the nervous regions that is important in fight‐or‐flight response (Creswell & Lindsay, 2014). In a pilot study, mindfulness‐based interventions in pregnancy have a positive effect on stress reduction, depression, and psychiatric disorders (Duncan & Bardacke, 2010). The results of systematic review studies on the effect of mindfulness have shown a favorable trend of stress reduction but no significant differences have been shown between groups. Due to the scarcity of trial studies, more research is needed to investigate the effect of mindfulness on stress and anxiety during pregnancy (Dhillon, Sparkes, & Duarte, 2017). Despite stress and anxiety risks for mother and fetus, limited stress reduction programs have been performed as a clinical trial during pregnancy, and only limited studies have focused on mind–body approach, advanced muscle relaxation, yoga, and meditation to reduce maternal stress to improve maternal health and pregnancy outcomes (Guardino, Dunkel Schetter, Bower, Lu, & Smalley, 2014).

The results of systematic review studies on the effect of mindfulness have shown a favorable trend and that no significant differences have been shown in the effect of mindfulness on anxiety, depression, and perceived stress. Due to the scarcity of trial studies, more research is needed to investigate the effect of mindfulness on stress and anxiety during pregnancy (Dhillon et al., 2017). The aim of the present study was to assess the effect of mindfulness based on stress reduction on the self‐efficacy and stress of pregnant women.

## 2 MATERIAL AND METHOD

This study was a randomized controlled trial study. Participants in this study consisted of pregnant women with a first 24‐ to 36‐week pregnancy referring to Abyek Comprehensive Health Services Centers. The Abyek Comprehensive Health Service Center is one of health centers in Qazvin province in Iran, where pregnant women referred to it.

Sample size with 95% confidence interval, 90% power and probability sample dropping, were estimated 35 per group.

$$n=\frac{\left(s_{1}^{2}+s_{2}^{2}\right){\left(z_{1-\frac{\alpha }{2}}+z_{1-\beta }\right)}^{2}}{{\left({\bar{x}}_{1}-{\bar{x}}_{2}\right)}^{2}}$$

**Figure 1:**
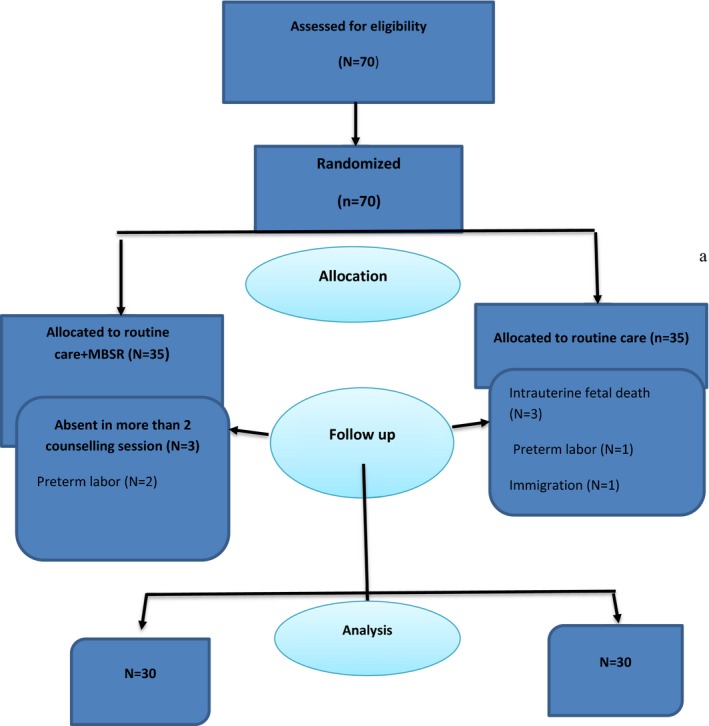
Consort flow diagram of study

In the intervention group, 3 women were excluded from the study due to lack of participation in more than two sessions of counseling sessions and 2 women excluded due to preterm labor. Moreover, in the control group, 3 women were excluded from the study due to preterm labor, intrauterine death, and immigration. Finally, the study was continued with 30 subjects in the control group and 30 in the intervention group (Figure 1).

### 2.1 Inclusion criteria

Being Iranian, fluent in Persian, first pregnancy, wanted pregnancy, 24‐ to 36‐week pregnancy, single fetus, and lack of pregnancy complications (diabetes, preeclampsia, etc).

### 2.2 Exclusion criteria

Nonparticipation in 2 full sessions of counseling, known medical conditions, psychological problems, abortion history, intrauterine death, preterm labor, participate in yoga classes, relaxation, and having cesarean section indications.

After approving study from Alborz university deputy research, written informed consent was obtained from all of mothers. The convenient sampling method was recruited. The samples were randomly assigned to each groups using randomized blocks: counseling (intervention) and without counseling (control). There were six probabilities for placing in blocks (BABA, BBAA ABBA, AABB, ABAB, and BAAB). At the beginning of the study, one of the blocks was selected and 4 participants were placed in that block, so that number A was assigned to the intervention group and number B belonged to the control group.

After determining the groups, before the onset of the study, pregnancy anxiety, self‐efficacy of coping with childbirth, and mindfulness questionnaire was completed in both groups.

### 2.3 Intervention

In the intervention group, pregnant women received 6 group counseling sessions (7‐person groups) twice a week, and each session lasted for 60 min in a lecture, question and answer, and role play based on the MBSR (Hauck, Fisher, Byrne, & Bayes, 2016). In addition to receiving weekly counseling, daily home‐based mindfulness methods were advised to the intervention group via telephone and social networks, and any questions were answered by the researcher. The researcher was a graduate student in midwifery counseling. The researcher had the necessary training in the content of the research sessions, the counseling days were reported weekly and the variable, to prevent information contamination. Finally, in order to observe the research ethics a handbook with the content of the consultation sessions was given to the control group. The content of the sessions was approved by the faculty members of the reproductive health, Obstetrics and gynecology and Psychiatric Departments of the Alborz University of Medical Sciences and implemented as follows:

Session 1: Greetings, explanation about mindfulness and its mechanism, explanation about the anatomy and physiology of the reproductive system of women and the natural process of labor, explaining how the social context affects the experiences and expectations at the time of delivery, explaining the active and expectant care model in the process of delivery, and assessing model of care which mothers intend to choose and empowering the pregnant mother to differentiate the benefits of both care models.
Session 2: Mindfulness breaths technique training, mindfulness and individual feelings, awareness of barriers to mindfulness exercises, expressing the importance of relaxing and comfortable moments during pregnancy and childbirth, and awareness of the obstacles to creating a feeling of goodness during this period.
Session 3: Repeating and practicing awareness, breathing, training for appropriate yoga positions for the fetus, relaxation and meditation techniques for reducing stress, using awareness breathing to control thoughts and feelings, training on using mindfulness in daily activity, awareness walking, sitting and moving.
Session 4: An Explanation of fearfulness with pregnancy, explaining the relationship between thoughts, fear and the impact of mindfulness of perceived fear, the use of problem‐solving skills to identify fears and find the right solution to overcome it, the experience of transition through thoughts and feelings, explanation and interpretation of the effect of mindfulness on the process of delivery, and practice and repetition of the use of mindfulness in daily activity.
Session 5: Practicing breathing technique, relaxation, sitting meditations, exercising suitable positions for the fetus, teaching the BRAIN technique (benefits, risks, alternatives, intuition, and nothing) to take appropriate interventions in pregnancy and childbirth.
Session 6: Meditation, awareness, breathing, talking about the needs of the neonate, explaining the symptoms of postpartum depression, using the BRAIN technique to make informed decisions, explaining breastfeeding and its benefits, identifying the needs of the neonate, postpartum activities, and talking about parenting challenges.

The data gathering instrument in this study were a demographic characteristic along with mindfulness questionnaire, anxiety, and self‐efficacy questionnaire.

A: Pregnancy‐Related Anxiety Questionnaire (PRAQ): This questionnaire was used to assess the concerns and fears in pregnancy and was created by Vandenberg in 1989 (Van den Bergh, 1990). In the psychometric evaluation of this questionnaire, Hizenk and his colleagues showed the correlation coefficients of the questionnaire as acceptable compared to Spielberger's anxiety questionnaire (Huizink, Mulder, de Medina, Visser, & Buitelaar, 2004). In Iran, it was also verified by Babanazari and Kafi (2008).

B: Mindfulness Questionnaire: The questionnaire was developed by Buchheld et al in 2001 (Buchheld, Grossman, & Walach, 2001). The Cronbach's alpha coefficient has been reported as 0.93. This questionnaire was verified and tested by Abolghasemi, Goolpour, Narimani, and Ghamari (2010).

C: Self‐efficacy in Coping with Childbirth questionnaire: It has been developed to measure maternal perception in adaptive labor pain; it measures the expected outcomes and predicted self‐efficacy. The validity and reliability of this questionnaire in Iran were measured by Khorsandi and colleagues in 2000 (Khorsandi et al., 2008).

### 2.4 Data analysis

In this research, data were analyzed using SPSS software version 16. Normality of data distribution was assessed by the Kolmogorov–Smirnov test. *t* Test was used to compare quantitative variables between the two groups. Comparison of qualitative variables was performed using chi‐square and Fisher's exact test. To compare pregnancy anxiety score, self‐efficacy of coping with childbirth, and mindfulness in two groups before, immediately, and 1 month after intervention, analysis of variance with repeated measures was used. Data analysis was performed on the basis of preprotocol analysis.

### 2.5 Ethical considerations

The present study was approved by the Ethics Committee with the code number (Abzums.Rec.1396.152) and recorded in the clinical trial system with the code number IR CT20150119020719N9. Before the onset of the study, the study objectives and the confidentiality of all information were explained to the participants. All participants signed informed consent to participate in the research and the researcher tried to consider all the moral rights of the research samples.

## 3 RESULTS

Demographic characteristics of pregnant women mentioned in Table 1.

**Table 1:** Demographic characteristic of pregnant women

Variable	Intervention	Control	*p* value
	Frequency (percent)	Frequency (percent)	
Mother's age (Mean ± *SD*)			
27 ± 5	30 (100)	0	.06*
24.5 ± 5	0	30 (100)	
Gestational age (week)			
30.4 ± 3.9	30 (100)	0	.09*
29.7 ± 3.8	0	30 (100)	
Education			
Under diploma	7 (23.3)	9 (30)	.15**
Diploma	14 (46.7)	18 (60)	
College	9 (30)	3 (10)	
Total	30 (100)	30 (100)	
Job			
Household	25 (83.3)	28 (93.3)	.32***
Employee	2 (6.7)	2 (6.7)	
Other	3 (10)	0 (0)	
Total	30 (100)	30 (100)	
Support for pregnant mothers			
Yes	27 (90)	28 (93.3)	.64**
No	3 (10)	2 (6.7)	
Total	30 (100)	30 (100)	

*T‐test; **Chi‐square; ***Fisher

The results of analysis of variance with repeated‐measures test showed that the length of time affects the total score of mindfulness (*p* = .001) and there is a significant difference between the two groups (*p* = .001) (Table 2).

**Table 2:** The trend of an effect the mindfulness on Pregnancy‐Related Anxiety and self‐efficacy of mothers in before, after, and 1 month after the intervention

Factor	Group	Before (Mean ± *SD*)	After (Mean ± *SD*)	One month after (Mean ± *SD*)	Muchly test	Repeated Measure Greenhouse–Geisser	
						Within group	Between group
Mindfulness	Intervention	29.7 ± 7.2	34.6 ± 5.1	37.8 ± 5.1	*p* = .02	*F* = 16.4 *p* = 0/001	*F* = 3,489.8 *p* = 0/001
	Control	32.7 ± 5.1	34.3 ± 4.8	35.3 ± 4.4			
Pregnancy‐Related Anxiety	Intervention	182.9 ± 74.2	154.5 ± 61.8	124.9 ± 45.5	*p* = 0/001	*F* = 4.6 *p* = 0/03	*F* = 1,058.7 *p* = 0/001
	Control	195.1 ± 42.9	187.9 ± 41.5	182.5 ± 41.7			
self‐efficacy in coping with childbirth	Intervention	104.2 ± 26.5	104.6 ± 25.8	105.3 ± 23.1	*p* = 0/001	*F* = 2/004 *p* = 0/1	*F* = 0/37 *p* = 0/69
	Control	103.06 ± 8.8	103.5 ± 8.5	105.7 ± 5.7			

The results of analysis of variance with repeated measures in assessing the changes in pregnancy anxiety score before, immediately after, and 1 month after the intervention showed that the length of time affects the anxiety score of pregnancy (*p* = .03) and that a significant difference was observed between the two groups in this regard (*p* = .001) (Table 2).

The results of analysis of variance with repeated measures in examining the changes in self‐efficacy scores before, immediately after, and 1 month after the intervention showed that the length of time did not affect the self‐efficacy score (*p* = 0. 1) and that there was no significant difference between the two groups (*p* = .69) (Table 2).

## 4 DISCUSSION

Mindfulness sessions had an effect on the reduction of anxiety. The results of this study are in line with those obtained in other studies. In a study, Yazdani Mehr and et al showed the effect of 8 sessions of cognitive–behavioral‐based mindfulness program on stress and depression in pregnant mothers (Yazdanimehr, Omidi, Sadat, & Akbari, 2016). The results of another 6‐session counseling showed anxiety reduction in the intervention and control group, but the anxiety reduction was reported more in the intervention group. In mentioning study, 30% of the participants in the control group stated that they participated in pregnancy yoga classes that may indicate the lack of a significant difference between the two groups (Guardino et al., 2014). The results of another study showed that pregnant women who participated in yoga classes and meditation for 3 hr once a week for 9 weeks increased mindfulness and decreased stress, anxiety, and their negative effects (Duncan & Bardacke, 2010). In a study in Australia, the effect of a mindfulness program in a randomized study on 32 pregnant women and in a nonrandomized study of 20 pregnant women was shown to reduce stress, anxiety, and depression, although there was no significant difference between the two groups (Woolhouse, Mercuri, Judd, & Brown, 2014). In a study, the mindfulness mobile app was designed to prepare the mental and emotional health of pregnant mothers and their husbands for childbirth and parenting transition. This app was designed to promote mental health and to resilience parents to childbirth and mental changes; however, it was not reported to be highly effective in people with severe psychological problems (Bakker, Kazantzis, Rickwood, & Rickard, 2016).

A qualitative study with the aim of explaining the experiences of mothers who had received the mindfulness during the pregnancy showed that many of the mothers considered mindfulness as a valuable preparation to confront the challenges of pregnancy, childbirth, and acceptance of parental roles, concerning stress, anxiety, and pain (Lönnberg, Nissen, & Niemi, 2018).

The results of this study showed anxiety reduction in the control group. It can be concluded that the three questionnaires of mindfulness, anxiety, and self‐efficacy by mothers simultaneously can lead to mother's awareness of their condition. Also, the progression of pregnancy in the control group increases the experience of pregnancy and stress reduction. In this study, participants were advised not to participate in other research and group classes during the course of the study. The study limitations were the inability to control information from other sources of information such as the Internet and attendance at health centers, which may reduce the stress score in the intervention group.

The results of this study showed that the self‐efficacy score was not significantly different immediately after intervention with before intervention; however, it was significant in 1 month after the intervention, which could be due to a higher decrease in stress in 1 month after the intervention. The results of a quasi‐experimental study showed the effect of mindfulness and empowerment model education on reducing stress and fear and improving self‐efficacy and delivery outcomes. The authors have declared the integration of mindfulness with a skill education to be a serious approach to improve delivery outcomes. It should be noted that the low number of the samples and the absence of control groups were subject to research limitation (Byrne et al., 2014). In the present study, the effect of mindfulness on anxiety and self‐efficacy was investigated. Mindfulness after intervention did not affect the self‐efficacy in coping with childbirth. According to the results of study, it is recommended that further research show the effect of blending mindfulness and skills‐based prenatal education program on self efficacy. In this intervention, none of the participants in the intervention group participated in childbirth preparation classes. The self‐efficacy score increased in 1 month after intervention compared to before and after intervention. Since maternal anxiety scores were also reduced in 1 month after, it is suggested that this variable be considered as one of the effective factors in mindfulness studies. It is also suggested that the mindfulness home work that is recommended to be done at home be emphasized and evaluated more reliable. So, in this area, it is possible to record the workouts at home by the participants.

## 5 CONCLUSION

This study showed that mindfulness reduces anxiety of pregnant mothers, and it is suggested that mindfulness programs be educated for healthcare providers and pregnant mothers so as to reduce maternal anxiety and improve pregnancy outcomes and delivery.

## CONFLICT OF INTEREST

None declared.

## AUTHOR CONTRIBUTION

Sara Esmaelzadeh is a supervisor and designed and performed the study; Masoomeh Zarenejad is a student who performed the study; Zahra Mehdizadeh designed and consulted about the proposal; Mansooreh Yazdkhasti designed and gave counseling about the content of the proposal and paper; and Mitra Rahimzadeh is a biostatistician and gave counseling about sampling and analysis of data.

## Data Availability

The data that support the findings of this study are available in the supplementary material of this article.
